# Knock-Down of a Novel snoRNA in *Tetrahymena* Reveals a Dual Role in 5.8S rRNA Processing and Generation of a 26S rRNA Fragment

**DOI:** 10.3390/biom8040128

**Published:** 2018-10-30

**Authors:** Kasper L. Andersen, Henrik Nielsen

**Affiliations:** 1 Biotech Research and Innovation Centre (BRIC), University of Copenhagen, Ole Maaløes Vej 5, DK-2200N Copenhagen, Denmark; 2 Department of Cellular and Molecular Medicine, The Panum Institute, University of Copenhagen, Blegdamsvej 5b, DK-2200N Copenhagen, Denmark; hamra@sund.ku.dk

**Keywords:** ribosome, ribosome biogenesis, pre-rRNA, *Tetrahymena thermophila*, ciliate, SNORD14, U14, sarcin-ricin loop

## Abstract

In eukaryotes, 18S, 5.8S, and 28S rRNAs are transcribed as precursor molecules that undergo extensive modification and nucleolytic processing to form the mature rRNA species. Central in the process are the small nucleolar RNAs (snoRNAs). The majority of snoRNAs guide site specific chemical modifications but a few are involved in defining pre-rRNA cleavages. Here, we describe an unusual snoRNA (TtnuCD32) belonging to the box C/D subgroup from the ciliate *Tetrahymena thermophila*. We show that TtnuCD32 is unlikely to function as a modification guide snoRNA and that it is critical for cell viability. Cell lines with genetic knock-down of TtnuCD32 were impaired in growth and displayed two novel and apparently unrelated phenotypes. The most prominent phenotype is the accumulation of processing intermediates of 5.8S rRNA. The second phenotype is the decrease in abundance of a ~100 nt 26S rRNA fragment of unknown function. Sequence analysis demonstrated that TtnuCD32 share features with the essential snoRNA U14 but an alternative candidate (TtnuCD25) was more closely related to other U14 sequences. This, together with the fact that the observed rRNA processing phenotypes were not similar to what has been observed in U14 depleted cells, suggests that TtnuCD32 is a U14 homolog that has gained novel functions.

## 1. Introduction

The maturation of rRNA in eukaryotes is a complex process taking place mainly in the nucleolus. A large precursor ribosomal RNA (pre-rRNA) is synthesized containing the 18S, 5.8S, and the 28S rRNAs (human nomenclature) with long external (5′ETS and 3′ETS) and internal (ITS-1 and ITS-2) transcribed spacers. The maturation of the pre-rRNA into mature rRNA species involves both extensive nucleotide modification, as well as a series of processing steps including cleavages by a endonucleases followed by trimming of ends by 5′ and 3′ exonucleases, where the non-coding internal and external spacers are removed [1,2,3]. The process involves a large set of transacting protein factors as well as up to ~200 small nucleolar RNAs (snoRNAs) [4]. SnoRNAs, with the exception of MRP RNA, are divided into two sub-groups based on the presence of conserved sequence motives: Box C (UGAUGA) and box D (CUGA) in box C/D snoRNAs and box H (ANANNA) and ACA in box H/ACA snoRNAs. The vast majority of box C/D snoRNAs guide site-specific 2′-O-methylations (2′-O-me) whereas the box H/ACA snoRNAs guide pseudouridylations (Ψ’s) of the pre-rRNA [5,6]. Both sub-groups complete their undertaking through base-pairing to the target molecule with their guide sequence(s). In box C/D snoRNAs the guide sequences are the nucleotides immediately 5′ of the 3′-terminal box D or the internal box D′. In box H/ACA snoRNAs the guide nucleotides are located on each side of internal loops situated in one, or both, of two stem-loop domains [7]. A few exceptional snoRNAs namely box C/D snoRNA U3, U14, U8, U22 and box H/ACA snoRNA sn30/U17 and snR10 are involved in the nucleolytic processing of pre-rRNA [8,9,10,11,12,13,14,15,16]. These ‘processing snoRNAs’ are believed to chaperone the pre-rRNA folding through base-pairing interactions, thereby preventing incorrect folding and/or creating the rRNA conformation competent for cleavage [1,4]. The majority of the 2’-O-me and Ψ modification guide snoRNAs are non-essential and can be deleted in cells with minor or undetectable effects [2,17]. Deletion or depletion of clusters of snoRNAs or specific individual snoRNAs, including both some modification guide snoRNAs and snoRNAs involved in the pre-rRNA processing, can each result in severe growth deficiencies, translation defects, or increased sensitivity to stress or antibiotics [4,18,19,20]. Finally, a few ‘processing snoRNAs’ are essential for cell viability. Of the latter group, snoRNA U3 is the most extensively studied. It is a large box C/D snoRNA and contain several additional conserved sequence boxes (GAC, A, A′, B). Through dynamic base-paired interactions with pre-rRNA, U3 is chaperoning the pre-rRNA folding and is involved in cleavages at three and five sites within the 5’ETS and ITS-1 in yeast and *Xenopus*, respectively [16,21]. SnoRNA U17 (yeast snR30) is one of two box H/ACA snoRNAs unambiguously demonstrated to be involved in pre-rRNA processing, and the only one to be essential. In absence of snR30, cleavages at three sites in pre-rRNA (A_0_, A_1_ in 5′ETS and A_2_ in ITS-1) are inhibited resulting in an abolishment of mature 18S rRNA accumulation [8,15]. SnoRNA U14 is unique among box C/D snoRNAs in vertebrates and yeast due to its dual function as a pre-rRNA chaperone involved in 18S rRNA processing, as well as a methylation guide snoRNA. Its functions are mediated by two conserved antisense elements, termed domain A and domain B, with complementarities to two sites in close proximity in the 18S rRNA secondary structure. The domains A and B are located in the 5′ and 3′ halves of snoRNA U14, respectively [10,22]. The domain A is responsible for U14 participation in pre-rRNA processing and the domain B is guiding the methylation event. Depletion of snoRNA U14 in yeast causes a defect in processing of pre-18S rRNA at sites A_1_ and A_2_ in 5′ETS and ITS-1, respectively, and deletion is deleterious [10]. Finally, the MRP RNA is an RNase P-related endoribonuclease that, in yeast, has been demonstrated to be involved in cleavage at site A_3_ in the ITS-1 [11]. 

**Figure 1:**
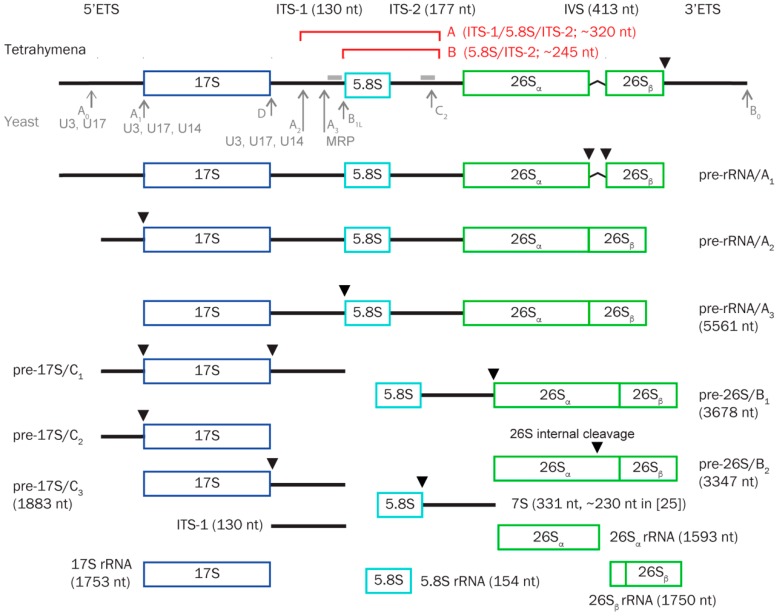
Pre-rRNA processing intermediates in *Tetrahymena*: Identified pre-rRNA species described in [25] are organized into a tentative processing scheme. *Tetrahymena* 17S corresponds to yeast/human 18S/18S, and 26S corresponds to yeast/human 25S/28S rRNAs. Inferred cleavage sites in *Tetrahymena* are indicated above the panels (black triangles) [25]. Endonucleolytic cleavages observed in yeast [1] are shown below the top panel for comparison (grey arrows). For cleavages involving recognized essential snoRNAs U3/snR17, U14/snR128, U17/snR30, and MRP the implicated RNAs are noted at the respective sites in grey. Brackets in red indicate the inferred positions of the two intermediates (A and B) that accumulate in TtnuCD32 KD cells. The approximate sizes of A and B are noted. Sizes of pre-RNAs based on the rDNA sequence (acc. no. X54512) and [25] are noted by the RNA when applicable. Two *Tetrahymena*-specific features are included, a self-splicing group I intron (IVS) in 26S and an internal processing site in 26S that process this RNA species into two fragments (26Sα and 26β) that both co-migrate with 17S in gel electrophoretic analysis. ETS and ITS: External and internal transcribed spacers. IVS: self-splicing group I intron. Grey bars indicate the positions of probes used in the northern blot analysis in Figure 3, Figures S3 and S4

Studies of pre-rRNA processing in the ciliate model organism *Tetrahymena* have to take its unusual genetic makeup into account. As most ciliates *Tetrahymena* has two structurally and functionally distinct nuclei. The micronucleus is the genetic nucleus and is only transcriptionally active during sexual reorganization (conjugation). The transcriptionally active macronucleus is derived from the micronucleus by several processes including deletion of segments, fragmentation, and amplification. Thus, the micronuclear genome is diploid and organized into five chromosome pairs, and the macronuclear genome is ~45 ploid and organized into 250–300 different fragments [23]. With respect to the rRNA genes, the single micronuclear copy is released from its parental chromosome during formation of the macronucleus and rearranged into a large (~21 kb) inverted repeat and amplified into ~9000 copies [24]. The primary transcript from the rRNA gene, its processing intermediates, and mature products 17S, 5.8S, and 26S_α_ and 26_β_ rRNAs (Figure 1) has been mapped by classical techniques including northern blot analysis and nuclease protection experiments in *Tetrahymena* [25] but is known in more detail from the mammalian and in particular the yeast model system [1,3,4]. The *Tetrahymena* rRNA distinguishes itself by the presence of a “hidden break” (cleavage site) in the 26S rRNA that cleaves 26S rRNA into two parts (26S_α_ and 26_β_) roughly the size of 17S rRNA [25]. This form of fragmented rRNAs incorporated into the mature ribosome is seen in a few other unicellular eukaryotes and is best known from *Trypanosoma brucei* that has the equivalent of 26S rRNA fragmented into six discrete sequences [26]. In addition, the *Tetrahymena* 26S rRNA harbors an autocatalytic group I intron that splices out as an early maturation step in *Tetrahymena* rRNA biogenesis [27].

Two of the essential snoRNAs have been identified in *Tetrahymena*, snoRNAs U3 [28] and U17 [8]. Additionally, a first snoRNA U8 candidate outside metazoans have been found in *Tetrahymena* [29]. Here, we investigate an unusual box C/D snoRNA (TtnuCD32) in *Tetrahymena*. Previously, the sequence of TtnuCD32 was reported as Tx-1 [30], and it was re-discovered and included in a general analysis of macronuclear ncRNAs of *Tetrahymena* as TtnuCD32 [29]. In this report, we demonstrate that genetic knock-out of TtnuCD32 compromises cell viability and conclude that TtnuCD32 is most likely an essential snoRNA. Further, we investigate predicted targets of TtnuCD32 box D and D’ guide sequences for methylations and conclude that such methylations appear not to exist. Then, we analyze the involvement in pre-rRNA processing by construction of a TtnuCD32 knock-down (KD) strain and demonstrate a role for TtnuCD32 in the maturation of the 5.8S rRNA as well as in the formation of a small fragment derived from the 26S large ribosomal rRNA. Both functions are, to our knowledge, novel rRNA processing phenotypes ascribed to a snoRNA.

## 2. Materials and Methods

### 2.1. Construction of TtnuCD32 Knock-Down Cell Lines

Clonal cell lines for TtnuCD32 knock-down were made in the strain SB210 background by targeted integration of the neo2 cassette in replacement of the endogenous TtnuCD32 gene. Two genomic elements, one upstream (5′: 714 bp) and one downstream (3′: 684 bp) of the TtnuCD32 gene were obtained by PCR on genomic DNA (gDNA). The 5′ and 3′ elements were cloned into the pBluescript KS(+) plasmid. Restriction enzyme sites (underlined) were added in the PCR. Oligos: kla22: CAG GGT ACC AAA ATC GCA AGG TAT GTA CAA AAT; kla23: CAG GGA TCC TTT GAA TGA TAA AAA TTA TGG GTT GA; kla24: CAG GGA TCC TGA ACT TAT AAT ATT TGT TGA ATT TCG; kla25: CAG GAG CTC TCA CCA ACA ATA AGC TTT TCA A). The paromomycin resistance cassette (neo2) was inserted between the two elements by sub-cloning. The endogenous TtnuCD32 gene including 83 bp upstream and 109 bp downstream was replaced by the neo2 construct by ballistic transformation using a Genegun (BioRad, Hercules, CA, USA) [31]. Subsequently, transformants were selected by paromomycin (Sigma, Merck KGaA, Darmstadt, Germany) supplemented medium initially at 175 µg/mL and subsequently raised gradually to 800–1200 µg/mL. Cells were kept at this concentration for three weeks to ensure that the maximal number of the ~45 chromosome copies in the *Tetrahymena* macronucleus was replaced with the TtnuCD32 knock-out construct. Single-cell sorting was performed to obtain clonal cell lines. 

The knock-out/knock-down cell lines were validated by Southern blot analysis. Genomic DNA (gDNA) was extracted from 2 mL of cell culture, washed once in 10 mM Tris-HCl, pH 7.5. The cell pellet was lysed by incubating at 37 °C for 1 h in 250 µL of lysis buffer (0.7 M NaCl, 20 mM Tris-HCl (pH 7.5), 20 mM EDTA, 2%SDS). DNA was extracted three times with PCI (phenol:chloroform:isoamyl alcohol 25:24:1) and precipitated with isopropanol before RNase A treatment and an additional PCI extraction. The gDNA was subsequently digested with BbsI (NEB, Ipswich, MA, USA), PCI extracted, and precipitated before 5 µg was resolved on a 0.7% agarose gel and transferred to a Hybond-N^+^ membrane (GE Healthcare, Chicago, IL, USA). The blot was analyzed by hybridization analysis using a probe made by random primer labeling of the 5′ flanking element of theTtnuCD32 mutant construct. 

### 2.2. TpnuCD32 Sequencing

To obtain the sequence of TpnuCD32, the homolog of TtnuCD32 from *T. pyriformis*, we extracted *T.pyriformis* gDNA and performed PCR using primers complementary to the 5′ end and 3′ end of TtnuCD32 and Taq polymerase (PCR oligos c90: GGA ATT CCA ACA TGA TGT ATA AAA CGC AT and c89: AAT CAG ACG ATT GGT AT). Then the same primers were used for dideoxy-sequencing of the PCR product from both ends applying a commercial kit (USB Affymetrix, Cleveland, OH, USA). The sequencing reactions were analyzed by 8% denaturing urea polyacrylamide gel electrophoresis (UPAG). 

### 2.3. Tetrahymena Cell Culture

Wild-type SB210 cells were maintained at 30 °C or at room temperature in NEFF media (0.25% proteose peptone, 0.25% yeast extract, 0.5% glucose, 30 µM FeCl_3_). TtnuCD32 KD and control cells, both containing the neo2 paromomycin resistance cassette, were maintained similar to SB210 cells but the medium was supplemented with 800–1200 µg/mL paromomycin. Cells for experimental analysis were transferred to medium without paromomycin and grown for 1–2 days before harvest at either the exponential growth phase (2–5 × 10^5^ cells/mL) or stationary phase (kept at maximum density ~1–2 × 10^6^ cells/mL for 20–24 h). 

Growth rates of TtnuCD32 knock-down cells and paromomycin resistant control cells at a non-coding locus were propagated in paromomycin containing NEFF media, transferred to NEFF media without paromomycin and grown overnight to approximately 2.5 × 10^5^ cells/mL. Cells were diluted to 1 × 10^5^ cells/mL and grown at room temperature while cell number was determined using a Neubauer cell chamber at approximately every hour during exponential growth 1–6 × 10^5^ cells/mL. The optimal exponential curve fitting the cell counts and the extra sum-of-squares *F* test to evaluate the *k*-values of the fitted curves were determined using the GraphPad Prism v7 software (GraphPad Software, Inc., La Jolla, CA, USA). From the *k*-value of the fitted curves the doubling times (T_2_) were calculated.

### 2.4. RNA Extraction and Northern Blot Analysis

For northern blot analysis and primer extension analysis nuclear RNA was isolated as previously described [29]. Whole cell RNA was isolated using TRIzol reagent (Invitrogen) and 5–20 µg RNA was resolved by 5% UPAG or 0.8% formaldehyde agarose gel electrophoresis. RNA was visualized by SYBR Gold (Invitrogen, (ThermoFisher Scientific), Waltham, MA, USA) or ethidium bromide staining and subsequently transferred to a Hybond-N^+^ membrane (GE Healthcare, Chicago, IL, USA) or BrightStar^+^ (Ambion, (ThermoFisher Scientific), Waltham, MA, USA). The probes were oligonucleotides ^32^P-labeled at their 5′-end in a standard T4 PNK reaction. The labeled oligos (kla21: 5′-TCA TCG AGG GAT GGC TTA AC for TtnuCD32; C642: 5′- TTG GAT GTT ATC CAG ATC TTA GAC AT for ITS-1; c632: 5′- TCT GGC GGC GAT TGC TCG ACC for ITS-2, c87: 5′- CTG CAA TTC GCA TTG CGT for 5.8S rRNA; kla49: 5′- CCT TCA GTC ATA ATC CA for rRNA 26S rRNA/T26 RNA) were hybridized over night to the membrane bound RNA at 42–50 °C in Denhardts hybridization buffer (4× Denhardts, 6× SSC, 0.1% SDS). Membranes were washed in wash buffer (3× SSC, 0.1% SDS) to low background. Alternatively, RNAs were detected with a random hexamer probe produced from linearized plasmid containing a 1.8 kbp genomic fragment including TtnuCD32 and TtnuCD33. Hybridization with random hexamer probe was done in 5× SSPE (0.75 M NaCl, 50 mM NaH_2_Po_4_ (pH 7.5), 5 mM EDTA, 5× Denhardt’s solution) at 65 °C. Washing was done in 1× and 0.1× SSPE supplemented with 0.1% SDS until the background was sufficiently low. For detection, membranes were exposed to a phosphor imager screen and scanned with a Typhoon 9400 scanner.

### 2.5. Primer Extension Analysis, Prediction, and Mapping of Methylated Nucleotides

Targets of TtnuCD32 were predicted using the SnoScan web service at http://lowelab.ucsc.edu/snoscan/ [32] by searching TtnuCD32 against *Tetrahymena* 17S, 5.8S, and 26S rRNA species extracted from *Tetrahymena* genome database (TGD www.ciliate.org) [33] using the yeast algorithm with a cut-off score of 2. Experimentally, 2′-O-me was detected by primer extension analysis of whole cell RNA at limiting dNTP concentration using M-MuLV reverse transcriptase (Fermentas (ThermoFisher Scientific), Waltham, MA, USA). Specifically, primer extensions were carried out at 1 mM, 0.04 mM, and 0.004 mM dNTP and compared [34]. For the 5′ end determination primer extension was carried out with non-limiting dNTP concentration (1 mM). The resulting DNA was gel electrophoresed through 7.5% UPAG next to the appropriate direct RNA sequencing reaction primed by the 1 pmol of the identical oligo (kla14: 5′- ATT CCA GCC GAT CCC GAG T for 17S rRNA; kla49: 5′- CCT TCA GTC ATA ATC CA for 26S rRNA; kla52: 5′- AGC CGA CAT CGA AGG AT for 26S rRNA).

### 2.6. Cluster Analysis

Sequences included in the analysis derived from the seed alignment for the SNORD14 family (RF00016) in the Rfam database [35]. Additional fly, mouse, and plant sequences were obtained from GenBank (AJ543993, NR028274, NR028275, NR028276, AF318017, AF318018, AF318016, AF318019) as well as TtnuCD32 (JF929905.1), TtnuCD25 (EF503642.1), and TtpuCD32 (MH888308). The cluster analysis was carried out using the phylogeny.fr website package (http://www.phylogeny.fr/) [36]. Multiple alignment was performed with TCoffee [37] and the alignment was curated with Gblocks [38] allowing for gaps and smaller size of final blocks. For phylogenetic analysis PhyML [39] was applied using the HKY85 substitution model. Branch support was analyzed by boot-strapping with 500 replicates. The resulting tree was visualized by TreeDyn [40].

## 3. Results

### 3.1. TtnuCD32 Structure and Conservation

**Figure 2:**
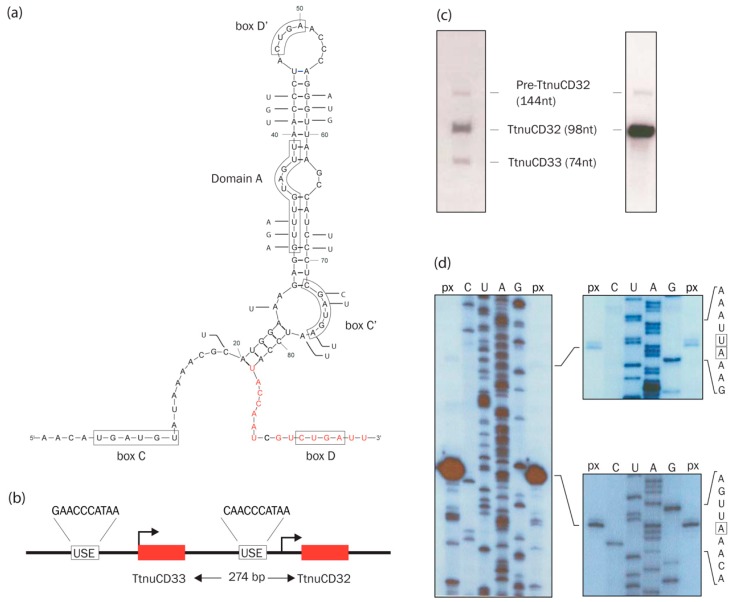
Mapping and structure analysis of the box C/D snoRNA TtnuCD32: (**a**) Secondary structure of TtnuCD32 derived by thermodynamic folding, structure probing, and comparative sequence analysis. Boxes C, C’, D, and D’, as well as a putative domain A are framed. Sequence variation between TtnuCD32 and the homolog from *T. pyriformis* (TpnuCD32) are noted by letters next to the sequence. Nucleotides involved in the non-canonical long interaction with 26S rRNA discussed in Figure 4 and Figure S2 are highlighted in red; (**b**) Genomic organization of the TtnuCD32 and TtnuCD33 genes. Sequences encoding the mature snoRNA are shown as red boxes. Upstream promoter elements (USE) are indicated; (**c**) Northern blot analysis of TtnuCD32 and TtnuCD33 on 10 µg nuclear RNA. The membrane in the left panel was hybridized with a probe produced from a 1.8 kbp genomic fragment containing both TtnuCD33 and TtnuCD32. For the right panel a TtnuCD32 specific probe was applied; (**d**) Mapping the TtnuCD32 5′ end. Primer extension analysis was performed (lanes marked px) and analyzed in parallel with a dideoxy-sequencing reaction of the cloned TtnuCD33/32 fragment (lanes marked C, U, A, and G) (left panel). The right panels show a magnification of the relevant parts of the autoradiogram with the inferred 5′-nucleotide framed in the sequence display.

TtnuCD32 was originally described as a relatively abundant snRNA of unknown function [30]. It harbors both a box C and D but is longer (98 nt) than the typical 60–80 nt box C/D snoRNA in *Tetrahymena* [29]. A secondary structure model (Figure 2a) was created based on structure probing experiments (not shown) and minimum free energy calculations by mfold (http://unafold.rna.albany.edu/?q=mfold/RNA-Folding-Form) [41] (Figure S1). Most of TtnuCD32 is folded into an elongated stem-loop structure interrupted by two small internal loops. TtnuCD32 has a D box (CUGA) at the 3′ end, and a near consensus box C (UGAUGU) toward the 5′ end. In box C/D RNAs, these usually base-pair to form a kink-turn motif that is bound by a small protein [4]. This interaction was not evident in our structure probing experiments and the structure is represented with unpaired ends to highlight the possibility of a long interaction with the proposed 26S rRNA target. Furthermore, internal boxes C’ and D’ could be identified (Figure 2a). Position 30–39 is able to base-pair with 17S rRNA similarly to domain A in snoRNA U14 from yeast and humans. The secondary structure model was further supported by analysis of the TtnuCD32 homolog from the distantly related *Tetrahymena pyriformis* by dideoxy sequencing of the RNA as well as of a genomic PCR-product. Compared to TtnuCD32 17 base substitutions were detected in the *T. pyriformis* sequence that did not include the 3′-most part of the sequence. The majority of the substitutions in TpnuCD32 resided in the stem regions, and several compensatory substitutions were observed. Interestingly, three substitutions that would shorten the potential interaction with 17S by three base-pairs were observed in the putative domain A, and three substitutions were detected in the internal box C’. The latter thus deviated further from the box C/C’ consensus sequence (UGAUGA) than what was observed for TtnuCD32. 

### 3.2. Genomic Organization, Expression, and Processing of TtnuCD32

TtnuCD32 is found in the genome at close distance from TtnuCD33, a canonical box C/D snoRNA (Figure 2b). Both have a snRNA/snoRNA-like upstream promoter element (USE) [28,29] situated 98 bp and 118 bp upstream of the mature RNAs, respectively, indicating independent transcription of the two RNAs. A northern blot analysis using a genomic fragment of 1.8 kb spanning the entire region revealed three RNA species (Figure 2c left panel). The two of lowest molecular weight corresponded to mature TtnuCD33 (74 nt) and TtnuCD32 (98 nt), respectively. The band of highest molecular weight RNA was identified as a TtnuCD32 precursor by application of a TtnuCD32 specific probe (Figure 2c right panel) and a primer extension analysis using a TtnuCD32 primer that revealed 5′-end of this 144 nt species (Figure 2d). Final proof of the precursor/product relationship of the two RNAs came from experiments that showed that the mature TtnuCD32 RNA but not the precursor could be labeled in a T4 kinase reaction after dephosphorylation and that it could be circularized with T4 RNA ligase and ATP (not shown). Both of these experiments imply that mature TtnuCD32 carries a 5′ monophosphate typical of a processed transcript.

### 3.3. TtnuCD32 is Unlikely to Function as a 2′-O-methylation Guide snoRNA

The majority of box C/D snoRNAs including snoRNA U14 guide site specific methylations of rRNA. They carry out their function by base-pairing to the target RNA with a stretch of nucleotides immediately upstream of the box D or D’ and direct methylation at a position base-paired to the nucleotide 5 nt upstream of the box D [7,42]. We applied the SnoScan software [32] to predict targets in rRNA of the box D and D’ associated guide sequences in TtnuCD32. The two highest scoring base-pair interactions and a third high scoring target with a particular interesting interaction are shown in Figure S2a. Target prediction (I) involved nucleotides in domain A and predicted a possible 2′-O-me in 17S rRNA at position C994. However, two mismatched base-pairs adjacent to the 2′-O-me site makes this a weak target prediction. Also, the stem structure of the central parts of TtnuCD32s, including nucleotides upstream of box D’ (Figure 2a), are supported by both structure probing, conservation, as well as compensatory base-substitutions in TpnuCD32 from *T. pyriformis*. This indicate that the region is not immediately free to guide a 2′-O-me. In agreement with the weak prediction, primer extension analysis did not show evidence of a 2′-O-me. Contrary, another methylation site (for an unrelated snoRNA) at A952 did give rise to a signal at the correct position (Figure S2b).

The second-best scoring target prediction (II) resembled a canonical box C/D snoRNA interaction in *Tetrahymena,* that generally are shorter than what observed for e.g., vertebrates [7,29]. However, even for a *Tetrahymena* box C/D snoRNA this interaction must be considered weak and with a centrally located mismatch in the base-pairing also this candidate can be considered unlikely to guide a 2′-O-me. The predicted interaction (III) between TtnuCD32 and 26S rRNA position 3031–3046 could guide methylation of U3036. However, similar to (I) and (II) the interaction does not seem adequate to guide a 2′-O-me. Strikingly, the interaction initially predicted by SnoScan could be extended across the box D to a non-canonical and unusually long (15 paired nucleotides in a stretch of 16) base-paired region, including the two TtnuCD32 terminal nucleotides on the 3′ side of the box D (Figure 2 and Figure S2a). For another study, a high-throughput method for detection of 2′-O-me (RiboMeth-seq [43]) carried out on *Tetrahymena* RNA from three different growth conditions [44] also did not provide any evidence for a 2′-O-me at position C994 (I) or the other two SnoScan predicted positions (II and III). Thus, we conclude that TtnuCD32 is unlikely to function as a 2′-O-me guide RNA.

### 3.4. TtnuCD32 is Critical for Cell Survival

**Figure 3:**
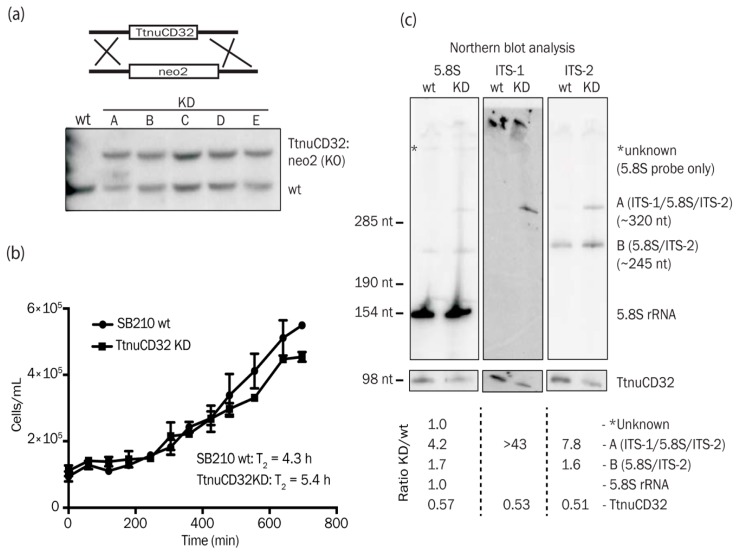
TtnuCD32 knock-down affects 5.8S rRNA maturation: (**a**) Upper part: Schematic illustration of the targeted substitution of the endogenous TtnuCD32 locus with the neo2 paromomycin resistance cassette. Lower part: Southern blot analysis demonstrating the partial replacement of the wild-type (wt) TtnuCD32 copies with the neo2 cassette in 5 mutant strains (A–E) creating the TtnuCD32 KD strains; (**b**) The growth curves averaged from two independent TtnuCD32 knock-down (KD) cell lines compared to two control cell lines. The doubling times (T_2_) for each cell type derived from the best fitted curve are given within the graph area. An Extra sum-of-squares *F*-test supported different growth curves as the best model to explain the datasets (*p* < 0.0001); (**c**) Northern blot analysis of the small molecular weight components of pre-rRNA processing in wild-type (wt) vs. TtnuCD32 KD cell lines (KD). Probes specific for 5.8S rRNA, ITS-1, ITS-2, and TtnuCD32 were applied as noted above the images. Sizes of the accumulating pre-5.8S species A and B was calculated based on the size/mobility relation of known RNAs visible by EtBr staining or northern blot analysis. Signals from the northern blot analysis were quantified and ratios KD/wt for rRNA species present on each panel are given below the images.

Next, we aimed at constructing a TtnuCD32 knock-out *Tetrahymena* strain. The strategy was to replace the endogenous gene for TtnuCD32 with the neo2 cassette conferring resistance against paromomycin as outlined in the upper panel of Figure 3a. However, after standard selection with paromomycin in multiple independent selections in order to replace the ~45 copies in the *Tetrahymena* cells we did not obtain a complete knock-out all wt chromosomes. Southern blot analysis on genomic DNA (gDNA) from individual clonal mutant strains all revealed only partial replacement (approximately 50%) of the ~45 endogenous loci with the neo2 cassette even following several months of continuous selection (Figure 3a, lower panel). Indeed, northern blot analysis confirmed the expression level of TtnuCD32 in KD cell lines to be ~50% of wt levels (see Figure 3c and Figure S2). Several constructs unrelated to the present work was selected in parallel, and all resulted in full replacement (fixation) after the paromomycin selection which is expected from a non-essential locus. Based on the resilience to being fully knocked out or efficiently knocked down, we conclude that TtnuCD32 is critical for cell survival and most likely is an essential snoRNA in vegetative growth. 

Next, we tested if any growth deficiencies of the TtnuCD32 KD strains could be observed. Since the TtnuCD32 KD strain was kept in paromomycin containing medium, we compared growth of two independent TtnuCD32 KD strains to two other independent clonal strains selected on paromomycin. Both control strains had an identical SB210 background and the neo2 cassette inserted next to a gene exclusively expressed during stress (the G8 RNA). Growth curves from the control and TtnuCD32 KD strains in a growth experiment carried out in NEFF medium at 24 °C can be seen in Figure 3b. The average doubling time for the control cells were 4.3 h, whereas the TtnuCD32 KD cell lines demonstrated a doubling time of 5.4 h. The experiment was carried out at temperatures ranging from 22–35 °C and they showed a similar tendency. Thus, cell lines deprived of TtnuCD32 grew slower than control cells, without being severely growth impaired.

### 3.5. TtnuCD32 Is Involved in Pre-rRNA Cleavages Adjacent to 5.8S rRNA

All known essential snoRNAs are involved in processing of pre-rRNA. Therefore, we tested the ribosomal processing pattern by northern blotting analysis of whole cell RNA separated on 0.8% denaturing formaldehyde agarose gels. No major differences between TtnuCD32 wt and KD were observed by ethidium bromide staining of the gel or northern blot analysis of the large rRNA products visualized by probes against ITS-1 and ITS-2 (Figure S3a). Accordingly, extended gel electrophoresis through a 5% denaturing polyacrylamide gel also did not reveal any differences between wt and KD large rRNAs judged by SYBR Gold visualization (Figure S3b). However, accumulation of RNA in the band representing small RNAs in the KD strain were evident when applying northern blot probes against both ITS-1 and ITS-2 (Figure S3a). Therefore, we analyzed the small RNA components on a 5% denaturing acrylamide gel using an oligo probe targeted against 5.8S rRNA (Figure 3c left panel). The 5.8S probe revealed clear differences between wt and TtnuCD32 KD strains related to the processing of the internal transcribed spacers. Besides the strong signal originating from 5.8S rRNA (154 nt) a signal corresponding to an RNA of an estimated size of 245 nt was observed (RNA B (5.8S/ITS-2)). This RNA was slightly more abundant in KD than in wt (ratio 1.7) cells. An additional signal corresponding to an RNA of 320 nt (RNA A (ITS-1/5.8S/ITS-2)) was almost exclusively observed in KD cells (ratio 4.2). The knock-down of TtnuCD32 to 57% of wt levels was verified by subsequent hybridization analysis of the filter (Figure 3c, left lower panels).

Two filters originating from a northern blot of a gel with samples run in parallel were then analyzed by ITS-1 and ITS-2 specific oligo probes, respectively (Figure 3c, middle and right panels). This confirmed that the 245 nt RNA B in addition to 5.8S rRNA included ITS-2 sequence and that the 320 nt RNA A included both ITS-1 and ITS-2 sequences. The accumulation ratio of wt to KD of the ITS-1/5.8S/ITS-2 species observed by ITS-1 and ITS-2 probes was even higher than what observed with the 5.8S probe (>43 and 7.8, respectively). Again, the knock-down of TtnuCD32 was confirmed on both filters. The sizes of the accumulating signals indicated that KD of TtnuCD32 resulted in a processing error giving rise to accumulation of 5.8S rRNA plus sequence from one or both ITSes. Compared to the published pre-rRNA sequence (5.8S = 154 nt, 5.8S/ITS-2 = 331 nt, and ITS-1/5.8S/ITS-2 = 461 nt) [45] this interpretation implies that both intermediates are products from cleavages within the ITS’es.

To ensure that the processing defects were not a result of features of TtnuCD32 clones unrelated to the experiment, we allowed the genomic copy number of the KD strains to revert towards full complement of ~45 wt gene copies by removing the selection media containing paromomycin and growing the cells for several generations. We analyzed RNA from wild-type cells (not paromomycin-resistant), a cell resistant to paromomycin by an unrelated tagged construct that should not interfere with growth or ribosomes processing and compared these to a TtnuCD32 KD strain kept in selective media, and the reverted/rescued TtnuCD32 KD strain grown without paromomycin. The two TtnuCD32 wild-type strains were very similar (Figure S4) and again accumulation of RNA B (5.8S/ITS-2) (ratio 1.8–3.4) and in particular RNA A (ITS-1/5.8S/ITS-2) (ratio 6.1–9.2) was observed in the KD strain (TtnuCD32 KD level to 40%). The rescued strain had recovered TtnuCD32 to 0.7 this was accompanied by accumulation of 5.8S/ITS species similar to wild-type levels with both 5.8S, ITS-2 and ITS-1 probes (Figure S4). Thus, we conclude that knock-down of TtnuCD32 correlate with accumulation of processing intermediates of 5.8S rRNA.

### 3.6. Abundance of a Small rRNA Fragment Correlates with TtnuCD32 Levels

**Figure 4:**
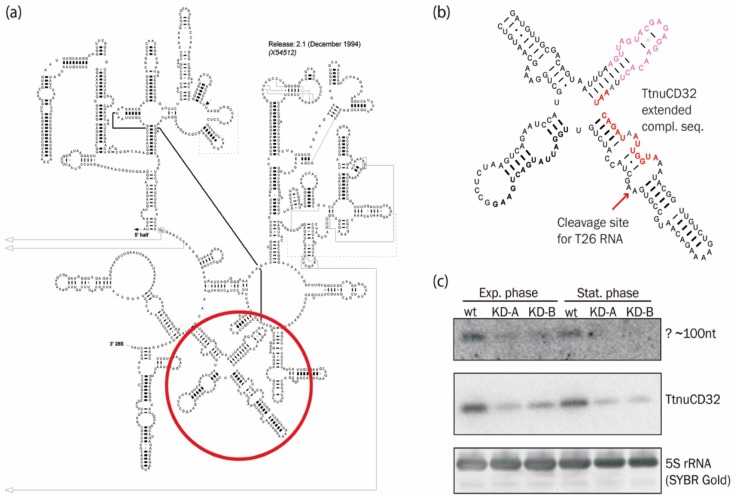
A small rRNA fragment derived from 26S rRNA in the vicinity of a putative non-canonical TtnuCD32 interaction site: (**a**) Secondary structure diagram of the 3′ end of *Tetrahymena* 26S rRNA. The structure was derived from the CRW web site (http://www.rna.ccbb.utexas.edu/) [47]. A red circle highlights position of the domain VI zoomed in on in (**b**); (**b**) Domain VI with the potential hybridization site of TtnuCD32 highlighted in red nucleotides, the sarcin-ricin stem-loop in purple and the sequence recognized by the probes used in (**c**) in bold letters. The 5′ end of the longest form of T26 RNA is marked by a red arrow; (**c**) Northern blot analysis of the 3′ end of 26S rRNA in RNA harvested from wt and two TtnuCD32 knock-down (KD) cell lines in exponential and stationary phase cells. Upper panel: An approximately 100 nt long RNA detected with a probe targeted against the 5′ end of T26 RNA (bold nucleotides in (**b**)). The middle panel shows the knock-down level of TtnuCD32 and the lower panel a SYBR gold staining of 5.8S rRNA as a loading control.

In Figure S2a we noted that 15/16 nt at the 3′ end of TtnuCD32 including box D was complementary to 26S rRNA at position 3031–3046, and that this remarkably long complementarity did not result in methylation at the predicted U3036. Two interesting notes emerged based on this putative TtnuCD32 target site: First, the target sequence overlap with the base of the stem containing the sarcin-ricin loop that is one of the longest universally conserved regions of rRNA. Secondly, the major part of the putative TtnuCD32 target is found in stem 95 (*S. cerevisiae* annotation) of 26S rRNA domain VI (Figure 4a). Interestingly, the opposite side of stem 95 harbors the 5′ end of T26 RNA, a 282 nt 3′-fragment of 26S that has been shown to be incorporated into the origin recognition complex (TtORC) involved in the initiation of rDNA chromosome replication in *Tetrahymena* [46] (Figure 4b). Since, TtnuCD32 has the potential to facilitate pre-rRNA cleavage, we hypothesized that TtnuCD32 could be involved in a cleavage resulting in the release of the T26 RNA from the remainder of 26S rRNA. An oligo probe targeted against the 5′ end of the T26 RNA was used in northern blot analysis of RNA from wt and TtnuCD32 KD cells during exponential growth and in stationary phase. With our setup, we failed to detect a signal corresponding to the 282 nt T26 RNA. However, a faint signal corresponding to a low molecular weight RNA of ~100 nt could be discerned and was found to correlate with the level of TtnuCD32. The signal was significantly decreased in TtnuCD32 KD cell lines (Figure 4c) and the decrease was more pronounced in stationary phase KD cells than in exponentially growing KD cells. This was the case even though the signal from TtnuCD32 was similar in wt cells between the two growth phases (upper panel), and the decrease in TtnuCD32 from the wt level to KD level was comparable in the two growth phases (middle panel). However, the RNA detected was significantly smaller than 26T RNA and its precise sequence and function, if any, is currently unknown.

### 3.7. TtnuCD32 Is a U14-Like snoRNA without the Cannonical Domain B

**Figure 5:**
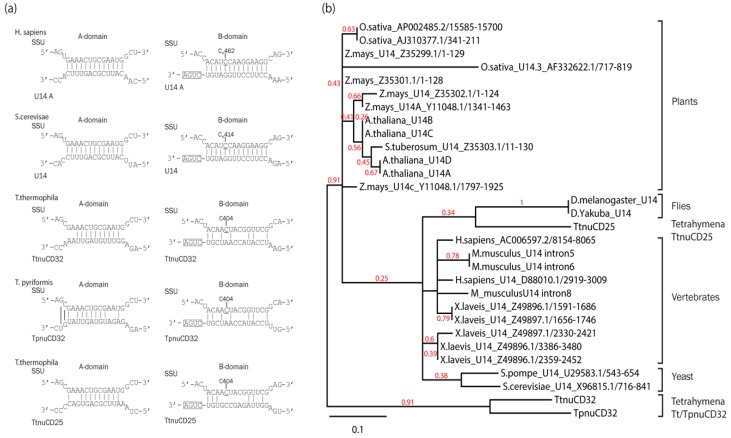
Comparison of TtnuCD32, TpnuCD32, and TtnuCD25 to snoRNA U14 from various species: (**a**) Interactions between the A and B box sequences of U14 snoRNA from yeast (*S. cerevisae*) and human (*H. sapiens*) as well as the corresponding regions in TtnuCD32 and TpnuCD25 from *T. thermophila* and *T. pyriformis* and their SSU rRNA targets; (**b**) A cluster analysis of U14 sequences from different eukaryotes as marked for each branch. Broader phylogenetic groups are noted at the side of selected clusters. Branch support from a bootstrap analysis with 500 replicates are shown at branches if above 0.25.

Two of the three known essential snoRNAs, U3 and U17 have previously been described in *Tetrahymena* [8,28,33]. In contrast, no U14 has been identified by bioinformatic or experimental analyses [29,33]. U14 is typically an 80–160 nt long box C/D snoRNA with two conserved sequence elements in addition to the canonical boxes C and D. The internal domain A is complementary to a site in the small subunit rRNA and involved in assisting maturation of this RNA [48]. Domain B functions similar to the box D of box C/D snoRNAs and is guiding the methylation of a ribose in the rRNA secondary structure close to the base-pairing interaction of domain A [22,48]. As indicated in Figure 2, TtnuCD32 contains an internal domain A-like sequence in a position similar to U14. To determine if TtnuCD32 could be a *Tetrahymena* snoRNA U14 homolog we compared the interactions between the domains A and B with their conserved target sites in the small subunit rRNA between TtnuCD32 and TpCD32 (the homolog from *T. pyriformis)* and known U14 snoRNAs from yeast and human. We also included TtnuCD25, another candidate for a *Tetrahymena* U14 snoRNA [29] (Figure 5a). Strikingly, none of the *Tetrahymena* U14 candidates could form a methylation guide interaction with the sequence in 17S rRNA targeted by U14 in yeast and humans. Neither domain B nor the 17S rRNA sequence surrounding the expected methylation site was conserved in the *Tetrahymena* examples. The domain A of U14 base-pairs with an invariant sequence in 17S rRNA. However, the putative domain A of TtnuCD32, TpnuCD32, and TtnuCD25 differ in sequence and their ability to base-pair to the target. TtnuCD32 can form 10 consecutive base-pairs. This is reduced to 7 base-pairs in TpnuCD32, while TtnuCD25 can form 9 consecutive base-pairings of identical sequence to that found in yeast and human. 

To further compare TtnuCD32, TpnuCD32, and TtnuCD25 to snoRNA U14, a full alignment and cluster analysis of 27 known U14 snoRNAs from plants, flies, vertebrates, yeasts, and the three *Tetrahymena* U14 candidates was carried out (Figure 5b and Figure S5). The cluster analysis resolved the known U14 snoRNA sequences into relevant clades. Thus, U14 snoRNAs sequences of plant, vertebrate, and yeast U14 snoRNAs were all assembled into monophyletic groups. Interestingly, *T. thermophila*, TtnuCD25 grouped with the fly U14 snoRNAs as a sister group to the other animal U14 snoRNAs. The fly U14 snoRNA, similar to TtnuCD25, TtnuCD32, and TpnuCD32, lack the otherwise conserved domain B responsible for guiding a 2′-O-me [49]. Nevertheless, the *T. thermophila,* TtnuCD32 and the *T. pyriformis*, TpnuCD32 were separated from the remaining sequences at the first node and formed their own group isolated from the remaining sequences with solid branch support. We tested several other alignment sequence compositions, alignment algorithms, stringencies, and substitution matrices for the cluster analysis and all gave a similar picture with the two snoRNAs TtnuCD32 and TpnuCD32 as a distinct group. The exact position of TtnuCD25 was variable and, in several analyses, it clustered within the plant group or in some together with a yeast U14 rather than together with the fly U14 snoRNAs. However, based on this analysis of the three *Tetrahymena* U14 candidates TtnuCD25 is related closest to known U14 snoRNAs from other organisms whereas, TtnuCD32 and TpnuCD32 can be considered more distantly related.

## 4. Discussion

Processing of pre-rRNA is a highly complex order of events involving a large number of protein factors such as endo- and exonucleases, GTPases, ATPases, and helicases, as well as hundreds of snoRNAs. Technical advances in recent years has made it possible to increase the understanding of the factors involved in ribosome biogenesis and their respective roles. The research in ribosome biogenesis has also been fueled by an increased understanding of genetic mutations in the ribosome and ribosome biogenesis machinery giving rise to severe diseases -the so-called ‘ribosomopathies’ [50]. Additionally, increased ribosome biogenesis is a hallmark of cancer and snoRNAs involved in pre-rRNA processing are required for tumorigenesis [51]. Accordingly, the ribosome biogenesis is emerging as a new target for anticancer treatments [52].

In this study, we have described a box C/D snoRNA, TtnuCD32, from *Tetrahymena thermophila* with two functions in rRNA processing not previously described. We failed to fully knock-out TtnuCD32 by ballistic transformation and subsequent selection of neo2 carrying KO chromosomes. Only partial knock-outs were obtained and continued expression of TtnuCD32 at ~50% of wt level was shown by northern blot analysis, also following an extended selection period (Figure 3 and Figure S3). Hence, we concluded that TtnuCD32 is a critical snoRNA for cell survival and most likely essential. TtnuCD32 is longer (98 nt) than the average box C/D snoRNA in *Tetrahymena*, but contain the canonical boxes C, C’, D, and D’ boxes. In addition, a sequence element with similarities to domain A of snoRNA U14 was identified. Transcription mapping showed that TtnuCD32 is transcribed as a 144 nt long precursor and subsequently processed to the mature 98 nt TtnuCD32. This is supported by the presence of a snRNA/snoRNA-like upstream sequence element (USE) believed to be a critical component of the promoter (Figure 2). Processing of pre-snoRNAs is common in vertebrates where the snoRNAs are situated within introns and processed by exonucleases to the mature RNA following mRNA splicing and debranching. Similarly, endo- and exonucleolytic processing of pre-snoRNAs are seen for polycistronic transcripts in yeast and plants [53]. We tried to replace the endogenous TtnuCD32 gene with a 3′ end RNA tagged version of TtnuCD32. The tagged construct replaced the wt genes in the genome and could be verified by Southern blot analysis, but no transcripts of the expected size could be detected. Instead, wt levels of wt sized TtnuCD32 was present in the cells (Figure S6). This indicates that the tagged TtnuCD32 was trimmed to a mature un-tagged form in the cell. Thus, it appears that TtnuCD32 is processed at both the 5′ and the 3′ ends suggesting the presence of a pre-snoRNA processing machinery in the *Tetrahymena* cell.

### 4.1. TtnuCD32 Is Involved in pre-rRNA Processing and Maturation of 5.8S rRNA

Since most box C/D snoRNAs, including the bi-functional and essential snoRNA U14, guide site specific 2′-O-me of rRNA, we investigated the modification guide potential of TtnuCD32. We identified three potential targets situated in rRNA regions that could base-pair with the D and D’ associated guide sequences in TtnuCD32. Furthermore, we tested the predicted targets experimentally (Figure S2). The target predictions were weak and accordingly, no evidence of modification was found by primer extension or RiboMeth-seq. Thus, it does not seem likely that TtnuCD32 is functioning in methylation of the rRNA. 

We demonstrated TtnuCD32 to impact growth rates and likely be essential for viability (Figure 3). The few essential snoRNAs known are all involved in pre-rRNA processing. Therefore, we investigated the processing pattern of pre-rRNA in TtnuCD32 KD cells. No major phenotype was robustly observed in analysis of larger rRNA products. However, we observed an aberrant pattern related to 5.8S rRNA formation. An intermediate containing approximately half of ITS-2 was slightly more abundant in KD cells and an intermediate that in addition contained a little more than half of ITS-1 was present in KD cells while barely detectable in wt cells (Figure 3c and Figure S4). These observations are consistent with a TtnuCD32-dependent processing defect at the primary cleavage site in ITS-1 followed by a perturbation of a secondary cleavage in ITS-2. In earlier studies of processing intermediates in *Tetrahymena* [25] (Figure 1) the primary cleavage leading to the maturation of the 5.8S rRNA was placed at the 5.8S 5′ end, but it cannot be excluded that this site should be placed within ITS-1 and perhaps coincides with a site known from more studied model organisms. This latter view is supported by the fact that knock-down of *Tetrahymena* Xrn2, responsible for trimming the 5.8S proximal nucleotides following ITS-1 cleavage at site E in mammalian cells, gave rise to accumulation of an ITS-1/5.8S precursor [54]. This indicates that the mature form of 5.8S is a result of ITS-1 cleavage followed by exonucleolytic trimming, also in *Tetrahymena*.

ITS-1 has previously been shown to be cleaved in a snoRNA dependent manner involving all of the known examples of essential snoRNAs (Figure 1), as well as the box C/D snoRNAs U8 and U22 described in animals and vertebrates, respectively [12,14]. However, mostly these ITS-1 cleavages have been linked to maturation of 18S rRNA and not to 5.8S rRNA. Experiments with U8 depletion in *Xenopus* oocytes [12] and in human cell lines [51] showed a decrease rather than an increase of a 5.8S/ITS-2 precursor upon snoRNA depletion. Thus, the pattern of accumulating pre-rRNA species were not identical to the ones observed in this study and the KD phenotype of TtnuCD32 appears unique in this aspect. In yeast and human the initial cleavages are typically internal in the ITS and are followed by trimming by exonucleases and/or secondary cleavages determining the mature ends of 18S, 5.8S and 26S rRNAs. In yeast the endonucleolytic cleavage of ITS-2 at C_2_ is initiated only following maturation of 5.8S 5′ end including cleavage at A_2_ and A_3_-sites (see Figure 1) [1,55]. If a similar scenario is relevant for *Tetrahymena* it is possible that the accumulating RNA B (5.8S/ITS-2) is not a direct result of TtnuCD32 KD but rather a consequence of the perturbed processing at the 5.8S 5′ end causing the accumulation of the most predominant pre-5.8S species: RNA A (ITS-1/5.8S/ITS-2). 

Notably, the remarkably long putative TtnuCD32 interaction target sequence overlap with the base of sarcin-ricin stem-loop (Figure 4b and Figure S2a III) and it is not clear if this interaction is responsible for the processing effect. The sarcin-ricin stem-loop is one of the longest universally conserved regions of rRNA and it has pivotal functions in translation, interacting with initiation, elongation, and release factors [56]. Additionally, the sarcin-ricin stem-loop has been shown to be essential for folding and assembly of the functional core of the ribosome [57]. It is possible that a snoRNA chaperone function is required for proper folding of this region in the *Tetrahymena* ribosome and perhaps also in other organisms. Early studies of human snoRNA U3 demonstrated protection against RNase T1 degradation of a stretch of nucleotides in human 28S rRNA overlapping the site predicted here to base-pair with TtnuCD32 [58]. The protected fragment was believed to be protected by a base-pairing interaction with snoRNA U3 but this proved to be inconsistent with subsequent sequence data from other organisms. An intriguing speculation is that the fragment was protected by interaction with a human TtnuCD32 homolog.

Consistent with the defect in pre-rRNA processing, we observed a decrease in growth rate of TtnuCD32 KD cells (Figure 3b). The relatively moderate effects on pre-rRNA processing and cell culture growth rates should be interpreted in relation to the modest TtnuCD32 KD levels that were obtained in these experiments (~50% of wt levels; Figure 3c and Figure S4). It is clear that a competition between impaired cell growth or cell death due to loss of drug resistance cassettes during amitotic division of the macronucleus and reduced growth rates due to TtnuCD32 depletion must be taking place in the cultures under paromomycin selection.

### 4.2. An Additional TtnuCD32 Facilitated rRNA Cleavage

We noted that TtnuCD32 could form an extraordinary long base-pair interaction with 26S that extended across the box D and included the two terminal nucleotides of TtnuCD32. The interaction is situated in the 26S rRNA secondary structure across from the 5′ end of the T26 RNA involved in replication of rDNA chromosomes in *Tetrahymena* [46]. This led us to speculate that TtnuCD32 could guide a 26S rRNA cleavage leading to the release of the T26 RNA. Therefore, we tried to detect the T26 RNA in exponential and stationary phase cells with both wt and KD levels of TtnuCD32. We failed to detect T26, but instead detected a low abundant RNA species that correlated with the level of TtnuCD32 (Figure 4c). The size of this RNA was estimated to approximately 100 nt which is considerably less than the 282 nt T26 RNA so our hypothesis was not supported. The 100 nt RNA was detected with a probe directed towards T26 RNA and, therefore, it shares sequence with this RNA. However, it remains to be characterized in details and we have currently no evidence of a function. Interestingly, a rapidly growing number of cleavage products of well-known ‘old RNAs’ such as tRNAs, snoRNAs, and Vault RNAs with independent functions have emerged the recent decade [59]. In addition to the T26 rRNA fragment, *Tetrahymena* as a model organism has pioneered studies involving tRNAs fragments [60,61,62]. Fragments of rRNA have been sporadically discovered [63,64] but have not been identified or studied to the same degree as e.g., tRNA fragments. This could partly be due to the wide-spread use of rRNA depletion steps before most high-throughput sequencing approaches combined with discarding of rRNA reads in libraries and the generally poor annotation of rRNA genes in databases. It is therefore an intriguing thought that additional rRNA fragments with functional importance exist. 

The presence of two apparently separate phenotypes correlating with knock-down of TtnuCD32 may seem puzzling. However, several examples of snoRNAs with more than one discrete function are known: examples include U3 and U14 as well as the snoRNAs processed into miRNA-like RNAs.

### 4.3. The Relation of TtnuCD32 to snoRNA U14

Identification of homologs of snoRNA U14 is complicated by the fact that domains A and B are functionally independent. This is most clearly observed in the fly where domain B in snoRNA U14 is absent. Instead, an unrelated box C/D snoRNA was predicted to guide methylation of the nucleotide targeted by U14 snoRNAs in other systems [49]. Thus, the absence of domain B does not preclude TtnuCD25 or TtnuCD32 as *Tetrahymena* snoRNA U14 homologs. According to the cluster analysis (Figure 5b), TtnuCD25 is closer related to U14 snoRNAs from other organisms than TtnuCD32. TtnuCD25 has a domain A with potential for base-paring with the relevant site in 17S rRNA (Figure 5a). The stretch of base-pairs is shorter than for TtnuCD32 but identical in sequence with that from yeast and human making this RNA the prime candidate for a U14 snoRNA in *Tetrahymena*. In contrast, TtnuCD32 was placed in a sister group to the range of U14 snoRNAs from different organism. Even yeast U14 that is known to diverge from canonical U14 snoRNAs in having an additional domain (Y domain) [65] was placed closer to plant and metazoan U14 sequences than TtnuCD32 (Figure 5b). Thus, our initial assumption of TtnuCD32 being the *Tetrahymena* U14 based on the primary sequence, including the presence of domain A-like sequence, is not supported by phylogenetic analysis. Furthermore, the lack of evidence for ribose methylation guiding and the processing phenotype of the KD strain that do not resemble U14 depletion in yeast, mouse, and *Xenopus* [10,22,48,66] together suggest that TtnuCD32 is not a *Tetrahymena* U14.

Several organisms have been shown to contain more than one copy of snoRNA U14. It is possible that TtnuCD32 and TtnuCD25 originate from a gene duplication followed by functional diversification resulting in a U14-like function of TtnuCD25 and TtnuCD32 acquiring a novel function in processing of pre-rRNA. In this respect, it would be of particular interest to study the phenotype of Ttnu25 KD strains. To our knowledge, the 5.8S processing phenotype observed with TtnuCD32 KD strains is novel. It would be of interest to characterize pre-rRNA processing in *Tetrahymena* in more detail to learn if the TtnuCD32 KD phenotype is related to a particular variation of pre-rRNA processing in *Tetrahymena* or is more general. Finally, the it will be of interest to study if a relationship exist between the ~100 nt 26S fragment that is found at lower levels in the TtnuCD32 KD strains compared to wt and the T26 fragment described in the literature to be a co-factor in rDNA replication [46]. The ~100 nt 26S fragment could be a 5′ end processing product of T26 and we speculate that TtnuCD32 in this way could be involved in coupling of rDNA transcription and pre-rRNA processing.

## Supplementary Materials

The following are available online at http://www.mdpi.com/2218-273X/8/4/128/s1, Figure S1. Predicted TtnuCD32 secondary structure. Figure S2. Analysis of putative TtnuCD32 methylation targets, Figure S3. Analysis of large RNA species in TtnuCD32 wild-type and KD cells. Figure S4. 5.8S rRNA maturation in TtnuCD32 KD and rescued cells. Figure S5. Multiple alignment of U14 snoRNAs and *Tetrahymena* U14 candidates. Figure S6. PP7-tagged TtnuCD32 is endogenously trimmed to wild-type length.
