# Supplementary material for: Knock-Down of a Novel snoRNA in Tetrahymena Reveals a Dual Role in 5.8S rRNA Processing and Generation of a 26S rRNA Fragment
Source: Biomolecules. 2018 Oct 30;8(4):128. doi: 10.3390/biom8040128 (PMC6315972; doi:10.3390/biom8040128)
Supplement: Supplementary file 1 [file biomolecules-08-00128-s001.pdf]

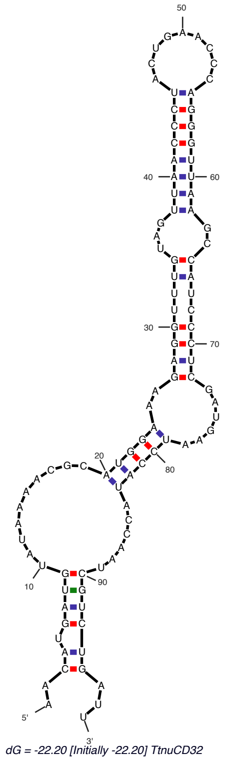

Figure S1. Predicted TtnuCD32 secondary structure: The secondary structure shown was predicted and the dG value of -22.2 calculated using the mFold web server (http://unafold.rna.albany.edu/?q=mfold/RNA-Folding-Form) [41].

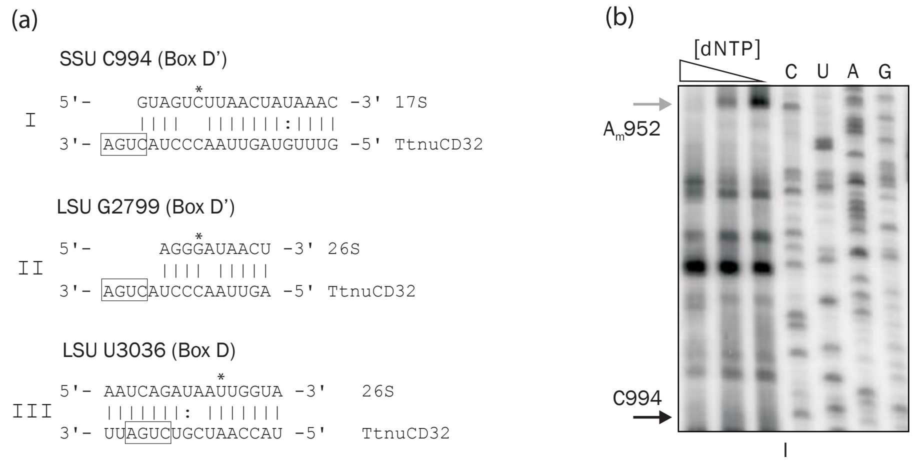

Figure S2. Analysis of putative TtnuCD32 methylation targets: **(a)** The SnoScan algorithm was applied to predict targets of TtnuCD32 box D and D' associated guide sequences. The SnoScan output from the highest scoring base-pairing interactions with rRNA are given. The D and D' boxes are framed, and predicted target nucleotides are indicated with asterisks above the base-paired nucleotides; **(b)** Experimental test of predicted nucleotide methylations shown in (a). Primer extension reactions with limiting concentrations of dNTP (1.0, 0.04 and 0.004 mM) were separated next to sequencing reactions of the target RNA. Predicted target of TtnuCD32 is indicated with a black arrow and the nucleotides position in SSU is noted. An experimentally detected methylation unrelated to TtnuCD32 is indicated by a grey arrow.

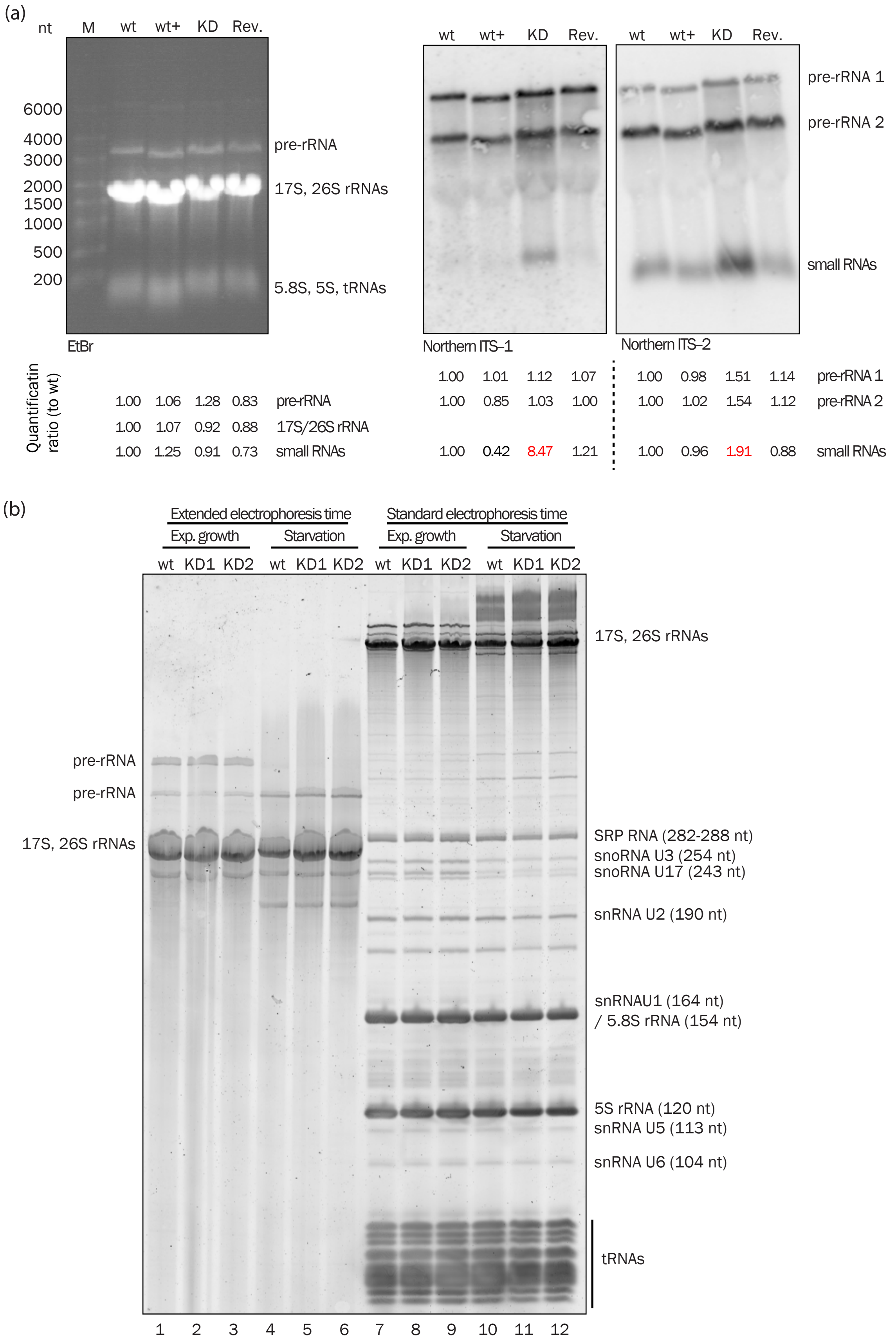

Figure S3 Analysis of large RNA species in TtnuCD32 wild type and KD cells: **(a)** Whole cell RNA was isolated from wild type (wt), paromomycin (Paro) resistant cells (resistance cassette inserted at a loci unrelated to rRNA biogenesis) grown in paromomycin (wt+), TtnuCD32 knock-down (KD) grown in Paro, and KD cells allowed to revert towards wt by removing Paro selection pressure for knock-out chromosomes (Rev.). The RNA was seperated on a denaturing formaldehyde agarose gel and stained with ethidium bromide (left panel) and analyzed by northern blotting against ITS-1 and ITS-2 (right two panels). Detected RNA species are noted on the sides and quantifications of each detected band relative to the same signal in wt cells are given below the images. Values of note are highlighted in red; **(b)** Isolated whole cell RNA from wt and two KD cell lines in exponential growth and under starvation conditions was seperated on 5% urea-polyacrylamide gel. The RNA was loaded at two different time points giving rise to lanes being seperated for an extended time (lane 1-6) and lanes being seperated for standard time (lane 7-12). The gel was stained with SYBR Gold for imaging and known RNA species are noted on the sides.

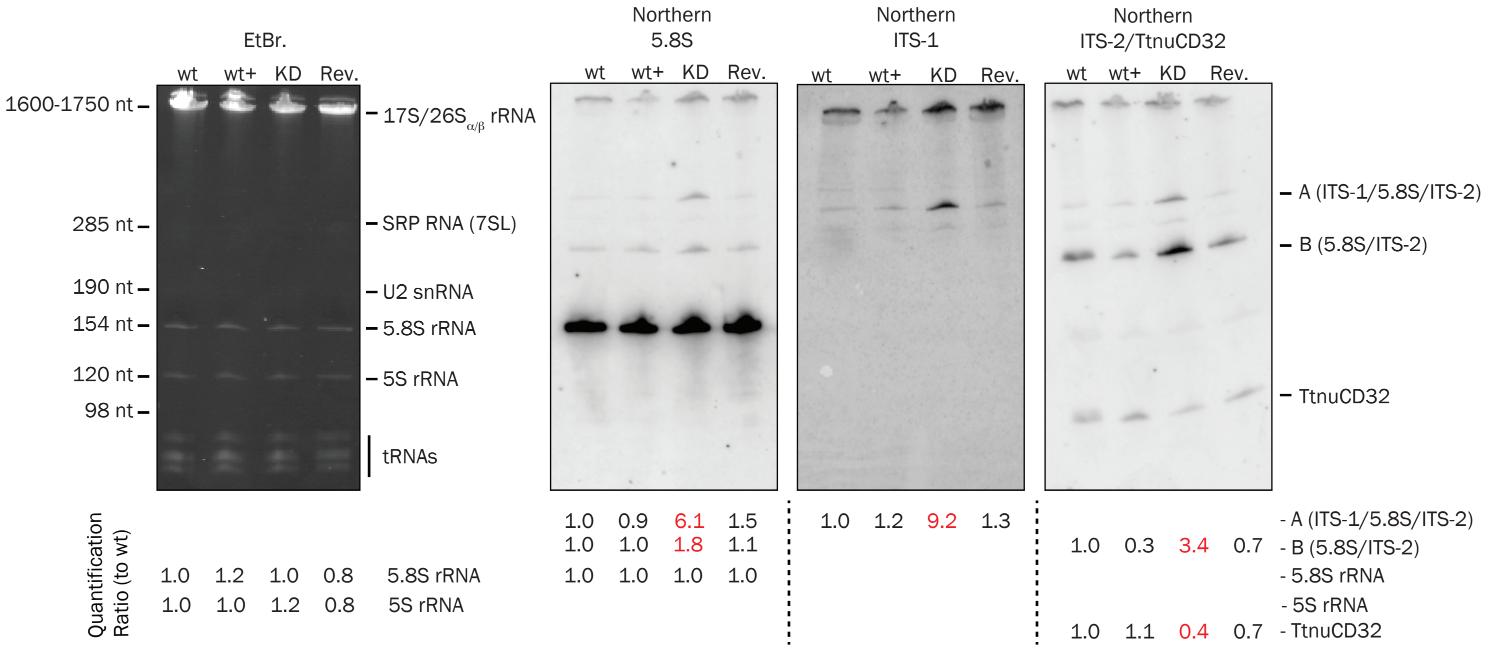

Figure S4. 5.8S rRNA maturation in TtnuCD32 KD and rescued cells: Small rRNA processing products in wild type cells (wt) compared to paromomycin resistant cells with a paromomycin (Paro) resistance cassette inserted at a loci not important for cell fitness or ribosome biogenesis grown with Paro (wt+), TtnuCD32 knock-down cell line grown with Paro (KD), and TtnuCD32 reverted /'rescue' cells (Rev.). Rescue cells are derived from KD cells but has been propagated without selection for numerous generations allowing for natural selection to reduce the number of genetic copies of the Paro cassette and replace them with wt TtnuCD32 containing copies. Left panel: Image of ethidium bromide (EtBr) stained gel. Molecular sizes derived from known RNA species visible by EtBr staining and the corresponding RNAs are noted on the sides. Right three panels: Northern blot analysis using oligo probes against 5.8S, ITS-1, or ITS-2 as noted above the panels. Signals from the EtBr gel image and the northern blot analysis were quantified and ratios KD/wt for detected rRNA species are noted below the images. Values found to differ between wt and KD cell lines are highlighted in red.

SnoRNA U14 multiple sequence alignment (TCoffee)

```
Scerevisiae_U14 UC-A-C------G-GUGAUG-AA---A----G--ACUGGU----UC-C-U
Xlaveis_U14-1   UC-A-C-----A--GUGAUG-AC--------U-----GGU---UUC-CA-
Xlaveis_U14-2   UC-A-C-----A--GUGAUG-AC--------U-----GGU---UUC-CA-
Hsapiens_U14A   UC-A-C-----U--GUGAUG-AU--------------GGUU--UUC-CA-
Hsapiens_U14B   UC-A-C-----U--AUGAUG-AU--------U-----GGU---UGC-CA-
Xlaveis_U14-3   UC-G-C-----U--GUGAUG-AA--------C--U-UGUU-----C-CA-
Xlaveis_U14-4   UC-G-C-----U--GUGAUG-AA--------U--G-UGAU----UC-CA-
Xlaveis_U14-5   UU-G-C-----U--AAGAUG-AA--------U--A-CGAU----UC-CA-
Zmays_U14-1     U--A-UGG-C-A--AUGAUG-AUGAAAGAU-A--A-GGCUUGUUUCUCA-
Zmays_U14-2     ---A-UGG-C-A--AUGAUG-UUGAA-GUU-A--AAGGCUUGUUUCUCA-
Osativa_U14-1   ---------C-A--AUGAUG-AUAAAU--U-U--AAGGCUUGUUUCUCA-
Zmays_U14-3     UGCA-UUG-C-AA-GUGAUG-ACAAAA--U-C--AAGGCUUGUUUCUCG-
Zmays_U14-4     UGCA-UUG-C-AA-GUGAUG-AUGAAG--U-C--AAGGCUUGUUUCUCU-
Osativa_U14-2   UGCA-UGG-C-AA-AUGAUG-CUAAAA--G-C--AAGGCUUGUUUCUCA-
Osativa_U14-4   U----------A--AUGAUG-CUAAAA--U-------------UUCUCA-
Zmays_U14-5     --CG-GUG-C-CCUAUGAUG-ACAAAA--U-C--AAGGCUUGUUUCUCU-
Stuberosum_U14  ---------C-UC--UGAUG-AUACAA--UUG--AAGGCUUGUUUCUCU-
Spompe_U14      UC-A-A---C-AG-GUGAUG-AA---A---------U--U----UC-CAU
TtnuCD25        ---A-AU-UC-AA-CUGAUG-AUUAAU---------UAGU---UUCUCU-
TtnuCD32        ---A-A---C----AUGAUGUAUAAAA---------CGCAUGGAA---A-
TpnuCD32        ---A-A---C----AUGAUGUAUAAAA---------CGUAUGGAUA-GA-
Dmelanog._U14   --------UUUUA-CUGAUG-AUUAA------------CU---UCAAC--
Dyakuba_U14     --------UG-AA-CUGAUG-AUUAC------------CU---UUAAC--
Athaliana_U14A  ------GGUU-AA--UGAUG-AUAA-A--U-CCAAAGGCUUGUUUUUCA-
Athaliana_U14B  --------UC-GA--UGAGG-AUAAGA--U-G--AAGGCUUGUUUCUCA-
Athaliana_U14C  --------UG--A--UGAGG-AUUAUA--U-U--AAGGCUUGUUUCUCA-
Athaliana_U14D  ---AAAGAUC--A-AUGAUG-AUAAAC--U-U--AAGGCUUGUUUCUCA-
Mmusculus_U14i5 UC-G-C-----U--GUGAUG-AU--------G-----GAU----UC-CA-
Mmusculus_U14i6 UC-G-C-----U--GUGAUG-AU--------G-----GAU----UC-CA-
Mmusculus_U14i8 UC-A-C-----A--AUGAUG-AU--------G--AAUGGU-----C-CA-
                                ** *
                                 Box C

Scerevisiae_U14 U-AA--CAUUCGCAGUUUCCACGGU---AG----GAGUACG-CUUACGAA
Xlaveis_U14-1   --AU--CAUUCGCAGUUUCUACC-----AG----AAA---G-CUU-----
Xlaveis_U14-2   --AU--CAUUCGCAGUUUCUACC-----AG----AAA---G-CUU-----
Hsapiens_U14A   ---A--CAUUCGCAGUUUCCACC-----AG----AAAG--G-UUU-----
Hsapiens_U14B   --GA--CAUUCGCAGUUUCCACC-----AG----AAAU--G-UUU-U---
Xlaveis_U14-3   --AAGCCAUUCGUAGUUUCCACC-----AG----AUGC-CG-AA--A---
Xlaveis_U14-4   --AAGCCAUUCGUAGUUUCCACC-----AG----AUGU-CU-CA--C---
Xlaveis_U14-5   --AAGCCAUUCGUAGUUUCCACC-----AG----AUGU-CG-UA--A---
Zmays_U14-1     ---A--CAUUCGCAGUAGCCGCC-U----A----AGA---G-CUUUCGC-
Zmays_U14-2     ---A--CAUUCGCAGUAGCCGCC-U----A----AGA---G-CUUUCGC-
Osativa_U14-2   U-AA--CAUUCGCAGUUGCCGCC-U----A----AGA---G-CUUUCGC-
Osativa_U14-4   ---A--CAUUCGCAGUUGCCGCC-U----A----AGA---G-CUUUCGC-
Zmays_U14-5     ---A--CAUUCGCAGUUGCCGCC-U----A----AGA---G-CUUUCGC-
Osativa_U14-2   U-AA--CAUUCGCAGUUGCCGCC-U----A----AGA---G-CUUUCGC-
Osativa_U14-3   U-AG--CAUCUGCAGUUACUGCC-U----A----AUA---G-CUUUCGC-
Zmays_U14-5     ---A--CAUUCGCAGUUGCCGCC-U----A----AGA---G-CUUUCGC-
Stuberosum_U14  A-AA--CAUUCGCAGUGGCCGCC-U----A----AGA---U-CUUUCGC-
Spompe_U14      UGAA--CAUUCGCAGUUUCCCC-------G----GAGC-GG-CAUACGAA
TtnuCD25        --AA--A-UUCGCAGUGACCCCCCUACUAGUCCGAAA---GGC-------
TtnuCD32        --GA--GGUUUGUAGUUAAC-CC-U----A----C--------UGAA---
TpnuCD32        --GA--GAU--GUAGUUAUG-UC-U----A----C--------UGAA---
Dmelanog._U14   --AC--CUUUUGCGGUUUCCACC-----AG----AAA---G-CUU-CG--
Dyakuba_U14     --AC--CUUUUGCGGUUUCCACC-----AG----AAA---G-CUU-CG--
Athaliana_U14A  A--A--CAUUCGCAGUGGCCGCC-U----A----AGA---G-CUUUCGC-
Athaliana_U14B  A-AA--CAUUCGCAGUGGCCGCC-U----A----A-G---G-CUUUCGC-
Athaliana_U14C  A-AA--CAUUCGCAGUGGCCGCC-U----A----A-A---G-CUUUCGC-
Athaliana_U14D  A-AA--CAUUCGCAGUGGCCGCC-U----A----A-A---G-CUUUCGC-
Mmusculus_U14i5 --AAACCAUUCGUAGUUUCCACC-----AG----AAGU------------
Mmusculus_U14i6 --AAACCAUUCGUAGUUUCCACC-----AG----AAAU------------
Mmusculus_U14i8 --AA--CAUUCGCGGUUUCCACC-----AG----AA---CG-CA--A---
                        *  *  **
                        Domain A

Scerevisiae_U14 CCCA----U-CGUUAGU-ACUCUCGGUGACCGCUCUU-CUU-UAGAGACC
Xlaveis_U14-1   --------UGC--CUGG-UG-----UUG---GCU---------AGUAACC
Xlaveis_U14-2   --------UGC---UGG-UG-----UUG---GCC---------UGUAACC
Hsapiens_U14A   --------UCC---UUAGUG-----UUG---GGU---------AAA--CC
Hsapiens_U14B   --------UCC---UUA-UG-----UUG---GCC---------AGU--UC
Xlaveis_U14-3   --------GGC---UGA-UG-----AUG---GCC------U--AGC-ACC
Xlaveis_U14-4   --------GAC---UUA-UG-----AUG---GUU------U--AUU-ACC
Xlaveis_U14-5   --------GAC---UCA-UG-----AUG---GCU------C----U-ACC
Zmays_U14-1     -C---AC--GCC--A--G-G-C---UCGAGAGCUUGUGCUGUUGA-AUCC
Zmays_U14-2     -C---CU--GCC--A--G-G-C---UUGAGAGCUUGUGCUGUUUA-AUCC
Osativa_U14-1   -C---CU--GCC--A--G-G-C---UUGAGAGCUAAUGCUGUUAA-UUCC
```

```
Zmays_U14-3     -C---CU--GCC--A--G-G-C---UUGAGAGGUAGUGCUGCCAA-AUCC
Zmays_U14-4     -C---CU--GCC--A--G-G-C---UUGAGAGCUAGUGCUGCCAA-AUCC
Osativa_U14-2   -C---UU--GCC--A--G-G-U---UUGAGAGCUAAUGCUGCUAA-UUCC
Osativa_U14-4   -C---CU--GCC--A--G-G-C---UUGAGAGCUAGUGCUGUUAA-AUCC
Zmays_U14-5     -C---CU--GCC--A--G-G-C---UUGAGAGCUAAUGCUGCAGA-AUCC
Stuberosum_U14  -C---UU-CGCC--A--G-G-C---UUGAGAGAUAAUGCUGCUUU-AUCC
Spompe_U14      CCCAAUUUUGCCGCAGA-UGUC--G-UG----------CAU--AG--UCC
TtnuCD25        -------------------G-C---UGUAG-GUUA---------G-AGCC
TtnuCD32        --------C-CC--A--GGG-----UUAA--GCCAUCCCUC---G-AUGA
TpnuCD32        --------C-CC--A--GAU-----GUAA--GCCAUUUCUC---C-UUGU
Dmelanog._U14   -------------------G-C---UUAAU-GAUGGU-CU----A-AGGC
Dyakuba_U14     -------------------G-C---UUAAU-GAUGGU-CU----A-AGGC
Athaliana_U14A  -C---UUUCGCC--A--G-G-C---UUGAGAGUUAAUGCUGUUUU-AUCC
Athaliana_U14B  -C---UUUCGCC--G--G-G-C---UUGAGAGCUUAUGAUGUUUUUAUCC
Athaliana_U14C  -C---UUUCGCC--A--G-G-C---UUGAGAGCUAAUGCAGCUUU-AUCC
Athaliana_U14D  -C---UUUCGCC--G--G-G-C---UUGAGAGUUAAUGCAGCUUUUAUCC
Mmusculus_U14i5 ---------GC---UG--UG-----UUG---GCU---------AGU-UCC
Mmusculus_U14i6 ---------GC---UG--UG-----UUG---GCU---------AGU-UCC
Mmusculus_U14i8 --------GGC---AG--UG-----UUG---GCA----------GUUACC


Scerevisiae_U14 UUCCUA-GG--AU-GUCUGAGUGA-------
Xlaveis_U14-1   UUCCUU-GG--AU-GUCUGAGUGA-------
Xlaveis_U14-2   UUCCUU-GG--AU-GUCUGAGUGA-------
Hsapiens_U14A   UUCCUU-GG--AU-GUCUGAGUGA-------
Hsapiens_U14B   UUCCUU-GG--AU-GUCUGAGUGA-------
Xlaveis_U14-3   UUCCUU-GG--AU-GUCUGAGCG--A-----
Xlaveis_U14-4   UUCCUU-GG--AU-GUCUGAGCG--A-----
Xlaveis_U14-5   UUCCUU-GG--AU-GUCUGAGCG--A-----
Zmays_U14-1     UUCCUU-GG--AU-GUCUGAGCCA---UA--
Zmays_U14-2     UUCCUU-GG--AU-GUCUGAGCCA---UA--
Osativa_U14-1   UUCCUU-GG--AU-GUCUGA--------G-C
Zmays_U14-3     UUCCUU-GG--AU-GUCUGACGCA-AUG-CA
Zmays_U14-4     UUCCUU-GG--AU-GUCUGAUGCA-AUG-CA
Osativa_U14-2   UUCCUU-GG--AU-GUCUGAUGCA-AU-GCA
Osativa_U14-4   UUCGUU-GA--AU-GCAUA------------
Zmays_U14-5     UUCCUU-GG--AU-GUCUGAGGGC--CGCCG
Stuberosum_U14  UUCCUU-GG--AU-GUCUGAGAUC-------
Spompe_U14      UUCCUUUGG--AU-GUCUGA-UGU-UG----
TtnuCD25        G----U--------GUCUGA-U---------
TtnuCD32        AUCCAU-ACCAAUCGUCUGAUU---------
TpnuCD32        UUCCAU-ACCAAUCGUCUGAUU---------
Dmelanog._U14   G----U----------CUGACU---------
Dyakuba_U14     G----U----------CUGACU---------
Athaliana_U14A  UUCCUU-GG--AU-GUCUGAAA-C--U----
Athaliana_U14B  UUCCUU-GG--AU-GUCUGA-----------
Athaliana_U14C  UUCCUU-GG--AU-GUCUGAAA----C----
Athaliana_U14D  UUCCUU-GG--AU-GUCUGAGAUCUUU----
Mmusculus_U14i5 UUCCUU-GG--AU-GUCUGAGCGA-A-----
Mmusculus_U14i6 UUCCUU-GG--AU-GUCUGAGCGA-A-----
Mmusculus_U14i8 UUCCUU-GG--AU-GUCUGAGUGA-C-----
                                 *

                  Domain B     Box D
```

Figure S5. Multiple alignment of U14 snoRNAs and Tetrahymena U14 candidates: Full multiple alignment of the three *Tetrahymena* U14 candidates TtnuCD25, TtnuCD32, and TpnuCD32 with twenty seven U14 snoRNAs from various species as noted for each line. The alignment was created using TCoffee [37] at the phylogeny.fr web server [36]. Box C and D as well as the domain A are underlined in the bottom line and noted under the alignment in red. Alignment of Domain B is highlighted in pink in all sequences. The cluster analysis for the final tree in Figure 5B was based on this alignment including a subsequent curation by Gblocks.

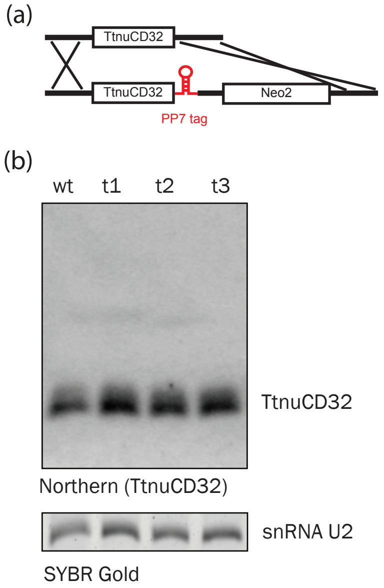

Figure S6. PP7-tagged TtnuCD32 is endogenously trimmed to wild type lenght: **(a)** Schematic illustration of the targeted replacement of the TtnuCD32 loci with a neo2 and tagged TtnuCD32 containing construct. The position of the PP7 hairpin tag in the 3'end of the mature TtnuCD32 RNA is highlighted (red); **(b)** Northern blot analysis of TtnuCD32 from wild type (wt) and three independent genetically modified strains having the wt loci fully replaced by the tagged TtnuCD32 construct in the genome (t1-t3) (upper panel). SnRNA U2 stained with SYBR Gold was used as loading control (lower panel).
